# Cytotoxic and Proapoptotic Activity of Sanguinarine, Berberine, and Extracts of *Chelidonium majus* L. and *Berberis thunbergii* DC. toward Hematopoietic Cancer Cell Lines

**DOI:** 10.3390/toxins11090485

**Published:** 2019-08-23

**Authors:** Anna Och, Daniel Zalewski, Łukasz Komsta, Przemysław Kołodziej, Janusz Kocki, Anna Bogucka-Kocka

**Affiliations:** 1 Chair and Department of Biology and Genetics, Medical University of Lublin, 4a Chodźki St., 20-093 Lublin, Poland; 2 Chair and Department of Medicinal Chemistry, Medical University of Lublin, 4 Jaczewskiego St., 20-090 Lublin, Poland; 3 Department of Clinical Genetics, Chair of Medical Genetics, Medical University of Lublin, 11 Radziwiłłowska St., 20-080 Lublin, Poland

**Keywords:** cytotoxicity, apoptosis, sanguinarine, berberine, *Chelidonium majus*, *Berberis thunbergii*, leukemia, anticancer

## Abstract

Isoquinoline alkaloids belong to the toxic secondary metabolites occurring in plants of many families. The high biological activity makes these compounds promising agents for use in medicine, particularly as anticancer drugs. The aim of our study was to evaluate the cytotoxicity and proapoptotic activity of sanguinarine, berberine, and extracts of *Chelidonium majus* L. and *Berberis thunbergii* DC. IC10, IC50, and IC90 doses were established toward hematopoietic cancer cell lines using trypan blue staining. Alterations in the expression of 18 apoptosis-related genes in cells exposed to IC10, IC50, and IC90 were evaluated using real-time PCR. Sanguinarine and *Chelidonium majus* L. extract exhibit significant cytotoxicity against all studied cell lines. Lower cytotoxic activity was demonstrated for berberine. *Berberis thunbergii* DC. extract had no influence on cell viability. Berberine, sanguinarine, and *Chelidonium majus* L. extract altered the expression of apoptosis-related genes in all tested cell lines, indicating the induction of apoptosis. The presented study confirmed the substantial cytotoxicity and proapoptotic activity of sanguinarine, berberine, and *Chelidonium majus* L. extract toward the studied hematopoietic cell lines, which indicates the utility of these substances in anticancer therapy.

## 1. Introduction

Sanguinarine and berberine are two of the most intensively investigated isoquinoline alkaloids in terms of their use in medicine. Sanguinarine is a benzophenantridine-type alkaloid occurring in *Papaveraceae*, *Ranunculaceae*, and *Berberidaceae* families. Berberine is a tertiary, protoberberine-derived alkaloid occurring in *Berberidaceae*, *Papaveraceae*, *Menispermaceae*, *Ranunculaceae*, and *Rutaceae* families. Due to their toxicity, sanguinarine and berberine play a defending role against viruses and fungi that are pathogenic towards plants [1,2].

Sanguinarine exhibits various pharmacological activities, including antibacterial [3], anti-inflammatory [4,5], anti-depressant [6], antinociceptive [7], antihypertensive [8], and antiplatelet [9] properties. It was previously demonstrated that sanguinarine inhibits acetylcholinesterase [10] and alfa-amylase [11], which broadens the potential clinical applications of this compound. Berberine is also an alkaloid with diverse biological activities [12], showing antimicrobial [13], anti-inflammatory, antioxidative [14], antidiabetic [15], cardioprotective [16], antidepressant [17], and neuroprotective effects [18].

The most intensively investigated of these is the anticancer activity of sanguinarine and berberine. Many pieces of evidence indicate that sanguinarine inhibits cell cycle and induce apoptosis in various types of cancer cells [19,20]. Sanguinarine induces apoptosis in receptor [21] and mitochondrial pathways [21,22,23,24]. In primary effusion lymphoma (PEL) cells exposed to sanguinarine, typical symptoms of the receptor-induced apoptosis were observed, including overexpression of DR5 receptors, activation of caspase-8, and truncation of BID protein. In turn, truncated BID protein mediated the mitochondrial pathway of apoptosis, which was evidenced by loss of mitochondrial membrane potential, release of cytochrome c to cytosol, and activation of caspase-3 and -9 demonstrated in PEL cells exposed to sanguinarine [21]. In high doses, sanguinarine also induces the death of cells via the process of necrosis [22,23]. The anticancer properties of sanguinarine also include inhibition of tumor invasiveness and angiogenesis through inhibition of matrix metalloproteinases activity and VEGF signaling [25,26,27,28].

Numerous studies have evidenced the anticancer activity of berberine [29,30]. Berberine was demonstrated to be non-toxic for normal cells and cytotoxic for cancer cells [31]. Berberine disrupts cell cycle, induces apoptosis, and inhibits angiogenesis [31,32,33]. Berberine enhances the radiosensitivity of cancer cells [34], but normal cells seem to be protected against radiation [35].

Clinical applications of sanguinarine and berberine are limited by their toxic effects [36]. In India, higher incidence of gall bladder cancer and epidemic dropsy was related to the consumption of mustard oil contaminated with sanguinarine [37,38]. The mechanism of cytotoxic and mutagenic activity of sanguinarine is an intercalation of DNA, binding to the tRNA molecules, causing induction of apoptosis and inhibition of oxidative phosphorylation and ATP synthesis [39,40,41,42,43]. Sanguinarine induces hepatotoxicity in animal models [44]. Berberine can intercalate DNA, but with much weaker effects than sanguinarine [45,46,47]. Berberine forms complexes with nuclear DNA and causes breaking in the double helix of DNA in a dose-dependent manner [48]. Berberine affects gene expression through binding to TATA boxes and the poly-adenine tails in mRNA [49]. This interaction is probably responsible for the neuroprotective effect of berberine in brain ischemia [50].

Despite the toxicity and mutagenicity of sanguinarine and berberine, the compounds are still extensively studied due to the possibility of synthesis of derivatives with reduced toxicity. In optimized doses, these alkaloids could exhibit potential therapeutic effects with limited side effects. A particularly important direction of research for this aspect is to determine the effect of the compound and mechanisms of action towards different types of cancer cells. Therefore, in this study we performed an assessment of cytotoxic and proapoptotic activities of sanguinarine and berberine on selected hematopoietic cell lines derived from various types of leukemia, including HL-60, HL-60/MX1, HL-60/MX2 (acute promyelocytic leukemia), J45.01 (acute T cell leukemia), U266B1 (myeloma), CCRF/CEM, and CEM/C1 (acute lymphoblastic leukemia). HL-60/MX1 and HL-60/MX2 cell lines are the multidrug-resistant derivatives of the HL-60 cells, therefore these cell lines were used to evaluate the effects of studied samples on cells resistant to anticancer treatment.

Previously a screening of alkaloid composition was performed for nine species: *Chelidonium majus* L., *Macleaya cordata* Willd., *Lamprocapnos spectabilis* (L.) Fukuhara, *Fumaria officinalis* L., *Glaucium flavum* Crantz, *Corydalis cava* (L.) Schweigg and Körte, *Berberis thunbergii* DC., *Meconopsis cambrica* (L.) Vig., and *Mahonia aquifolium* (Pursh) Nutt. [51]. The highest amount of sanguinarine was demonstrated in *Chelidonium majus* L. and the highest amount of berberine was found in *Berberis thunbergii* DC. [51]. Therefore, extracts of these two species were also included in this study for evaluation of their cytotoxic and proapoptotic activity toward selected hematopoietic cell lines.

Plant extracts are very complex mixtures of various compounds, which could exhibit antagonism or synergy of biological activity. The application of plant extracts in pharmacotherapy has a higher risk of side effects than pure compounds, the advantage of which is a more predictable therapeutic effect. In this study, we evaluated the cytotoxicity and proapoptotic activity of both pure alkaloids, sanguinarine, and berberine, as well as the extracts prepared from plants containing high amounts of these alkaloids, *Chelidonium majus* L. and *Berberis thunbergii* DC., respectively.

## 2. Results

### 2.1. Sanguinarine, Berberine, and Chelidonium majus L. Extract Exhibit Cytotoxic Activity against Hematopoietic Cell Lines

Cytotoxicity of sanguinarine, berberine, and extracts of *Chelidonium majus* L. and *Berberis thunbergii* DC. toward HL-60, HL-60/MX1, HL-60/MX2, CCRF/CEM, CEM/C1, J45.01, and U266B1 cell lines was evaluated by determination of IC10, IC50, and IC90 doses in trypan blue staining test (Table 1). Sanguinarine exhibited the highest cytotoxic activity against all study cell lines, with low variability in the IC10, IC50, and IC90 doses between individual cell lines. The strongest cytotoxic effect of sanguinarine was observed toward HL-60/MX2 cells. CCRF/CEM and U266B1 cell lines were the least sensitive to the exposure to this compound (Table 1).

Berberine was cytotoxic to all tested cell lines, but significantly less so than sanguinarine. The cell viability of the HL-60/MX2 cells exposed to berberine did not fall below 50% despite exposure to the maximum concentrations possible to be obtained. Therefore, IC50 dose could not be determined for this line due to the poor cytotoxicity of the compound. The maximum dose to which HL-60/MX2 cells were exposed (250 μM) was marked as IC50 with an asterisk (IC50*). The IC90 values of berberine were determined only for the CCRF/CEM cells at130 μM and J45.01 cells at200μM. For the remaining cell lines, the IC90 dose could not be determined due to insufficient cytotoxicity of berberine even at the maximum possible dose (250 μM) when the viability of the U266B1, CEM/C1, HL-60, and HL-60/MX1 cell lines did not fall below 90%. For this reason, the maximum dose to which the cells were exposed was marked as IC90*. The most potent cytotoxicity of berberine was found toward CCRF/CEM, J45.01, and HL-60/MX1 cells. The HL-60/MX2 line was the least sensitive to the compound (Table 1).

The *Chelidonium majus* L. extract showed differentiated cytotoxic effect depending on the study cell line. The strongest cytotoxic effect of *Chelidonium majus* L. extract was exerted on the J45.01, CCRF/CEM, and CEM/C1 cells, and the cells of the HL-60 and HL-60/MX2 lines were the least sensitive to this extract (Table 1).

The extract of *Berberis thunbergii* DC. showed no cytotoxicity toward any of the cell lines tested. The maximum concentration of this extract administered in the cytotoxicity tests was 145.5 μg/mL, because in this concentration the DMSO content in the assay is equal to 0.5%, which is the maximum concentration not having a cytotoxic effect on the cells. The viability of exposed cells remained in the range of 89–95% for all tested cell lines. In this respect, IC10, IC50, and IC90 were not determined for *Berberis thunbergii* DC. extract.

### 2.2. Sanguinarine, Berberine, and Chelidionium majus L. Extract Affect the Expression of Genes Related to Apoptosis

Gene expression analysis considered 18 genes associated with apoptosis and was performed in cells exposed to IC10, IC50, and IC90 doses of sanguinarine, berberine, and *Chelidonium majus* L. extract as well as to *Berb4eris thunbergii* DC. extract in 145.5 µg/mL concentration using a real-time PCR with relative quantification method. Analysis of these gene expression data revealed differences in levels of expression in exposed cells compared to controls (non-exposed cells) (Figures S1–S8). Standard deviation, logRQ, and mean values are presented in Tables S1–S10 in Supplementary Materials.

#### 2.2.1. U266B1 Cell Line

Significant overexpression of *BAK1*, *BNIP3*, and *CASP9* was observed in U266B1 cells after exposure to sanguinarine, berberine, and *Chelidonium majus* L. extract. For IC10 doses, the highest gene expression modulatory activity was exhibited by sanguinarine, but in higher doses of tested samples, the most affecting expression of studied genes was berberine. A higher dose of berberine and a lower expression of *BCL2*, *BIK*, *BNIP2*, and *CASP3* were observed (Table 2, Figure S1).

#### 2.2.2. CEM/C1 Cell Line

Exposure to IC10 doses of sanguinarine and *Chelidonium majus* L. extract caused an increase in expression of the majority of studied genes, with *BAK1* and *MCL1* reaching logRQ > 1. For IC50 and IC90 doses of tested samples, the highest expression was observed for *BAK1*, *BNIP3*, and *CASP9* in cells exposed to berberine. All genes (except *BIK*) were upregulated in cells exposed to sanguinarine in IC10 dose, but in cells exposed to IC90 dose of this alkaloid these genes (excluding *BAK1* and *MCL1*) were downregulated (Table 2, Figure S2).

#### 2.2.3. CCRF/CEM Cell Line

Sanguinarine at IC10 dose caused a significant increase in expression of all studied genes, with *BAK1*, *BCL2*, *BCL2L2*, *BNIP3*, and *CASP9* reaching logRQ ≥ 2. In the case of IC50 and IC90 doses of this compound, upregulation of these genes was diminished. The distinctively high expression of the *TP53* gene was observed in cells exposed to IC50 and IC90 doses of sanguinarine, berberine, and *Chelidonium majus* L. extract (Table 2, Figure S3).

#### 2.2.4. HL60 Cell Line

Sanguinarine, berberine, and *Chelidonium majus* L. extract did not cause substantial changes in gene expression, excluding *BCL2L2* and *TP53* genes. *BCL2L2* and *TP53* were highly expressed in cells exposed to IC10 of *Chelidonium majus* L. extract and sanguinarine, respectively. Berberine caused notable downregulation of *TP53* in each dose of exposure (Table 2, Figure S4). 

#### 2.2.5. HL60/MX1 Cell Line

This cell line was characterized by a high level of differentiation in gene expression levels after exposure to sanguinarine, berberine, and *Chelidonium majus* L. extract. The influence on gene expression levels was larger in cells exposed to IC10 doses of all tested samples and to all doses of berberine. *BAK1*, *BCL2*, and *TP53* were the most upregulated genes in cells exposed to IC10 dose of berberine. For IC50 dose, berberine caused a significant decrease in *TP53* expression level. Significant downregulation of this gene was also caused by sanguinarine in IC50 and IC90 doses (Table 2, Figure S5).

#### 2.2.6. HL60/MX2 Cell Line

Berberine in IC10 dose caused a pronounced increase in expression of all studied genes, but for IC50 dose, the high expression remained only for *BAX*, *BIK*, *CASP3*, *MCL1*, and *TP53*. Sanguinarine at IC50 concentration induced upregulation of *BAD*, *BCL2L1*, *BCL2L2*, *BNIP2*, *CASP8*, *CASP9*, *PMAIP1*, and *TP53*, but for IC50 and IC90 doses of this compound these genes were significantly downregulated (Table 2, Figure S6).

#### 2.2.7. J45.01 Cell Line

Upregulation of *BNIP3*, *BCL2*, *CASP9*, and *BIK* was observed in J45.01 cells after exposure to sanguinarine, berberine, and *Chelidonium majus* L. extract in IC10, IC50, and IC90 concentrations (Table 2, Figure S7).

After exposure to sanguinarine in IC10 dose, the highest expression of almost all studied genes (excluding *MCL1* and *TP53*) was observed in CCRF/CEM cells compared to other cell lines. In turn, the highest expression of *MCL1* and *TP53* was demonstrated in CEM/C1 and HL-60 cells, respectively. The highest induction of genes associated with apoptosis by IC90 dose of sanguinarine was also observed in the CCRF/CEM cell line. In HL60/MX1 and HL60/MX2 cells, sanguinarine caused the lowest alterations in the expression of the studied genes. In these cell lines, the highest increase in expression of studied genes was observed after exposure to IC10 of berberine. Expression of examined genes in cells exposed to a higher concentration of berberine and extracts of *Chelidonium majus* L. and *Berberis thunbergii* DC. was differentiated across studied cell lines. Exposure to *Berberis thunbergii* DC. extract (145.5 µg/mL) caused upregulation of *B2M* and downregulation of *BAD* and *BNIP2* in all studied cell lines (Table 2, Figure S8).

### 2.3. Clustering and PARAFAC Analysis

**Figure 1:**
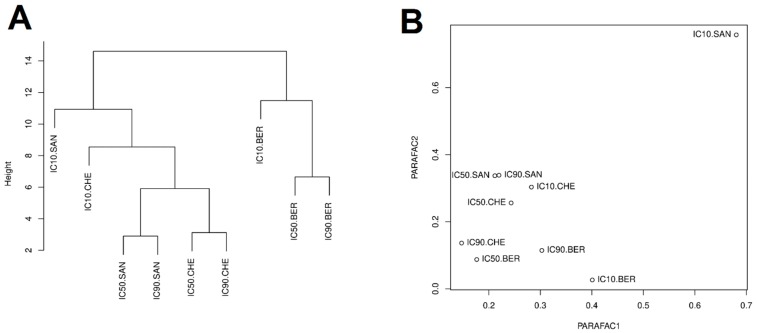
Distribution of similarities in apoptosis-associated gene expression in response to the IC10, IC50, and IC90 doses of sanguinarine, berberine, and *Chelidonium majus* L. extract, evaluated by the clustering analysis with Euclidean distance (**A**) and the PARAFAC analysis (**B**). Note: IC10.SAN = IC10 concentration of sanguinarine; IC50.SAN = IC50 concentration of sanguinarine; IC90.SAN = IC90 concentration of sanguinarine; IC10.BER = IC_10_ concentration of berberine; IC50.BER = IC50 concentration of berberine; IC90.BER = IC90 concentration of berberine; IC10.CHE = IC10 concentration of *Chelidonium majus* L. extract; IC50.CHE = IC50 concentration of *Chelidonium majus* L. extract; IC90.CHE = IC90 concentration of *Chelidonium majus* L. extract.

In order to assess the distribution of similarities in the doses of tested compounds in overall gene expression, a combined chemometric analysis of all values of gene expression changes obtained after exposure of the tested cell lines to sanguinarine, berberine, and *Chelidonium majus* L. extract at the IC10, IC50, and IC90 doses was performed (Figure 1).

Clustering with Euclidean distance revealed two subgroups, one of them containing sanguinarine with *Chelidonium majus* L. extract doses, and the other covering berberine doses (Figure 1A). This indicates that cellular responses in studied gene expression after exposure to berberine differ from the response to exposure to sanguinarine and *Chelidonium majus* L. extract.

PARAFAC analysis was performed on a tensor consisting of expression data for 18 genes, 7 cell lines, and 9 doses (IC10, IC50, and IC90 for sanguinarine, berberine, and *Chelidonium majus* L. extract). The PARAFAC univariate analysis explained only 28.3% of the variance, so bivariate analysis was performed, explaining 50.4% of the variance. The obtained results allowed the isolation of IC10 concentration of sanguinarine as an outlier (Figure 1B).

**Figure 2:**
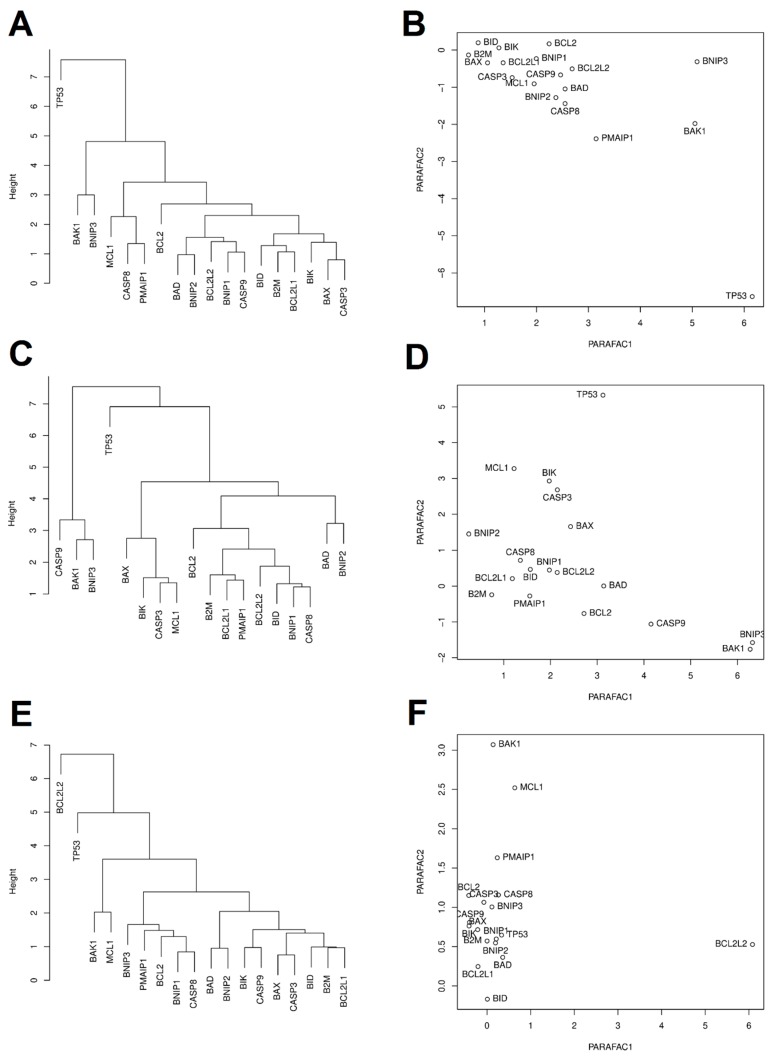
Distribution of similarities in expression of 18 apoptosis-associated genes after exposure to sanguinarine (**A**,**B**), berberine (**C**,**D**), and *Chelidonium majus* L. extract (**E**,**F**), evaluated by the clustering analysis with Euclidean distance (**A**,**C**,**E**) and the PARAFAC analysis (**B**,**D**,**F**). Note: IC10.SAN = IC10 concentration of sanguinarine; IC50.SAN = IC50 concentration of sanguinarine; IC90.SAN = IC90 concentration of sanguinarine; IC10.BER = IC10 concentration of berberine; IC50.BER = IC50 concentration of berberine; IC90.BER = IC90 concentration of berberine; IC10.CHE = IC10 concentration of *Chelidonium majus* L. extract; IC50.CHE = IC50 concentration of *Chelidonium majus* L. extract; IC90.CHE = IC90 concentration of *Chelidonium majus* L. extract.

Clustering analysis based on Euclidean distances and PARAFAC analysis were performed to assess similarities between the expression of 18 examined genes in the studied cell lines after the exposure to sanguinarine, berberine, and *Chelidonium majus* L. extract, (Figure 2). After exposure to sanguinarine, expression of *TP53* significantly differ from the expression of other studied genes. After exposure to berberine, expression of *TP53*, *BAK1*, and *BNIP3* differed from the expression of other studied genes. After exposure to *Chelidonium majus* L. extract, expression of *BCL2L2*, *BAK1*, and *MCL1* was different from the expression of other studied genes (Figure 2).

**Figure 3:**
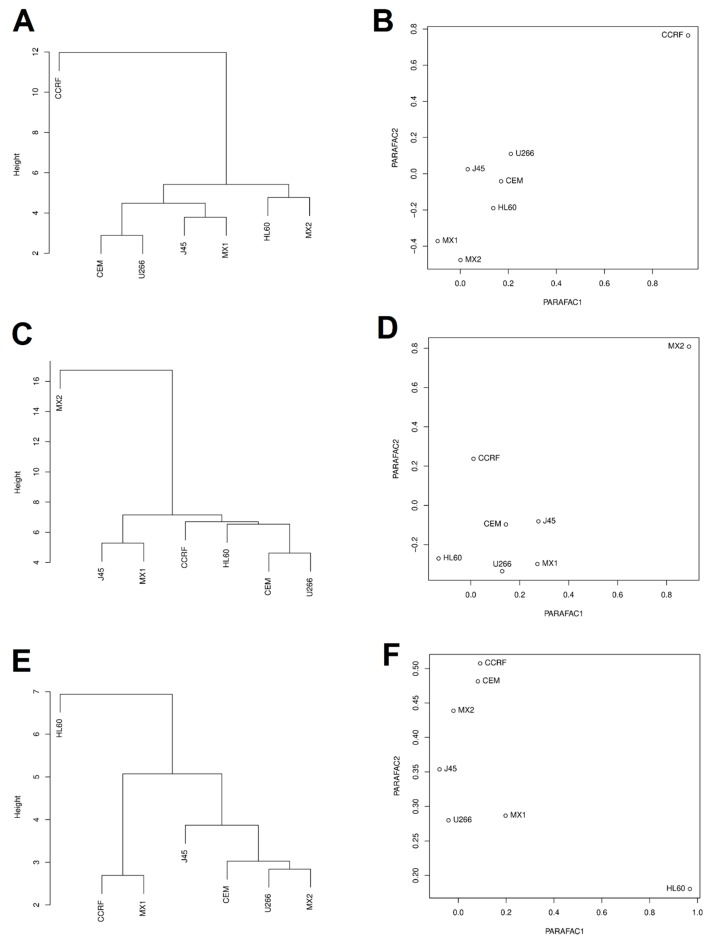
Distribution of similarities in cell lines response in expression of 18 apoptosis-related genes after exposure to sanguinarine (**A**,**B**), berberine (**C**,**D**), and *Chelidonium majus* L. extract (**E**,**F**), evaluated by the clustering analysis with Euclidean distance (**A**,**C**,**E**) and the PARAFAC analysis (**B**,**D**,**F**). Note: IC10.SAN = IC10 concentration of sanguinarine; IC50.SAN = IC50 concentration of sanguinarine; IC90.SAN = IC90 concentration of sanguinarine; IC10.BER = IC10 concentration of berberine; IC50.BER = IC50 concentration of berberine; IC90.BER = IC90 concentration of berberine; IC10.CHE = IC10 concentration of *Chelidonium majus* L. extract; IC50.CHE = IC50 concentration of *Chelidonium majus* L. extract; IC90.CHE = IC90 concentration of *Chelidonium majus* L. extract.

In order to assess the biological response to exposure to sanguinarine, berberine, and *Chelidonium majus* L. extract, the similarity of cell lines in changes in expression of studied genes was also assessed by Euclidean clustering and PARAFAC analyses. CCRF/CEM, HL60/MX2, and HL60 cell lines differed from other cell lines after exposure to sanguinarine, berberine, and *Chelidonium majus* L. extract, respectively (Figure 3).

## 3. Discussion

In this study, the cytotoxicity and proapoptotic activity of sanguinarine, berberine, and extracts of *Chelidonium majus* L. and *Berberis thunbergii* DC. were investigated. IC10, IC50, and IC90 doses were determined in trypan blue staining tests. The influence of these doses on expression of 18 apoptosis-related genes was evaluated.

Sanguinarine exhibited high cytotoxic activity against all studied cell lines. IC50 concentrations of sanguinarine were established toward HL60 cells in previous studies, showing values of 0.37 µM [52] and 1.02 µM [53]. The value obtained in our study was similar and was 0.6 µM.

Inhibitory concentrations received for *Chelidonium majus* L. extract indicated the cytotoxicity of this extract against all tested cell lines. The molar concentration of sanguinarine in the IC50 doses of *Chelidonium majus* L. extract (0.114–0.686 μM) was similar to the IC50 dose of the sanguinarine solution (0.1–1.05 μM) [51]. These findings suggest that sanguinarine is the main compound responsible for the cytotoxic activity of *Chelidonium majus* L. extract, but sanguinarine does not occur in the highest amount in this plant [51,54].

Our research confirmed the cytotoxic activity of *Chelidonium majus* L. extract, as previously evidenced in HL60 cells [55]. The results broaden the wide spectrum of evidence supporting the potential clinical application of preparations containing *Chelidonium majus* L. [56].

Berberine also exhibited cytotoxic activity against all examined cell lines, however it was weaker than sanguinarine. IC10, IC50, and IC90 doses of berberine were significantly higher than determined for sanguinarine and was characterized by a high variation between individual cell lines. Due to low cytotoxicity, we were not able to determine the IC50 dose of berberine for HL-60/MX2 cells and IC90 doses for HL-60, HL-60/MX1, HL-60/MX2, CEM/C1, and U266B1 cell lines. Cytotoxic activity of berberine toward U266B2 cell line was previously evaluated by Hu and collaborators using Cell Counting Kit-8 [57]. The authors reported a statistically significant reduction in cell viability after 48 h exposure to 40–160 μM of berberine. In our study, reduction in cell viability was achieved after exposure to higher berberine concentration (IC50 = 240.45 μM), which probably was a result of the shorter exposure time in our study (24 h). The cytotoxicity of berberine toward CCRF/CEM cell line was investigated by Efferth and collaborators in a 96-h model using MTT test [58]. The IC50 dose of berberine determined in the cited study was equal to 26 μM. The IC50 dose of berberine determined in the current work was higher and amounted to 80.00 μM, which was probably caused by the application of the shorter 24-h model.

In our study, the cytotoxic activity of berberine was evaluated for the first time in relation to J45.01, HL60/MX1, and HL60/MX2 cell lines.

The demonstrated cytotoxicity of berberine toward tested cells suggests the potential activity of *Berberis thunbergii* DC. extract, which contained a relatively high amount of berberine [51]. However, this extract at the maximum dose of 145.5 μg/mL did not cause a decrease in the cell viability of any of the tested cell lines. The probable explanation of this phenomenon is that the content of berberine in the *Berberis thunbergii* DC. extract is insufficient to induce a cytotoxic effect, which is supported by relatively high values of IC10, IC50, and IC90 concentrations (25.15 μM–250.45 μM) determined for berberine.

In the next step of the study, the influence of sanguinarine, berberine, and extracts from *Chelidonium majus* L. and *Berberis thunbergii* DC. on the expression of 18 genes associated with apoptosis was tested in hematopoietic cell lines. The similarities between administered doses and the response of the cells after 24 h exposure to the tested samples were also investigated.

Exposure to sanguinarine, especially in IC10 dose, caused the strongest upregulation of studied genes associated with apoptosis. Upregulation of *BAK1* and *BNIP3* was the most frequently observed. In CCRF/CEM cell line, all studied genes were upregulated with logRQ > 0.5 after exposure to sanguinarine. Berberine also exhibited an ability to raise expression of studied genes, but to a lower extent than sanguinarine. All studied genes were upregulated in HL-60/MX2 cells after IC10 berberine treatment. The expression of *TP53* was differentiated across studied cell lines exposed to IC50 of berberine. In CEM/C1, CCRF/CEM, and HL-60/MX2 cells *TP53* was upregulated, but in other cell lines the downregulation of this gene was demonstrated. This indicates diverse *TP53*-mediated responses of studied cells after treatment with berberine. Upregulation of *BAK1* and *BCL2L2* was the most often observed effect in studied cell lines after treatment with *Chelidonium majus* L. extract.

PARAFAC analysis shows that IC10 dose of sanguinarine was an outlier among other doses of tested samples. Exposure to sanguinarine at this dose induced interesting changes in gene expression, which seem to be more unambiguous in interpretation and more beneficial than those induced after exposure to higher doses of the compound. Sanguinarine in IC10 doses induced an increase in *TP53* gene expression. This is a beneficial effect because the promotion of neoplastic transformation cancer is associated with a reduced expression of the *TP53*. These findings support evidence that sanguinarine has a strong antiproliferative effect on cells with *TP53* gene dysfunction [21]. In the case of cells exposed to the IC90 dose of sanguinarine, an adverse decrease in the expression of *TP53* was observed.

The presented results show that the effect of *Chelidonium majus* L. extract on the expression of genes related to the apoptosis process is very similar to the effect of sanguinarine in the IC10 dose. This suggests similarities in the mechanism of apoptosis induction between sanguinarine and *Chelidonium majus* L. extract. Upregulation of *CASP8* and *CASP3* observed in cells exposed to IC10 of sanguinarine and IC10, IC50, and IC90 of *Chelidonium majus* L. extract indicated induction of apoptosis in the extrinsic pathway. The intrinsic apoptosis is probably prevented due to the accumulation of sanguinarine close to the outer side of the inner mitochondrial membrane during its stimulation. Sanguinarine neutralizes the effects of stimulation of the mitochondrial membrane, inhibits the synthesis of ATP, and breaks the oxidative phosphorylation [40]. An increase in *CASP9* expression observed in cells exposed to *Chelidonium majus* L. extract suggests the intrinsic course of apoptosis, probably conjugated with the extrinsic apoptosis pathway.

Another effect that clearly indicates promotion of the process of apoptosis in cells exposed to both *Chelidonium majus* L. extract and low sanguinarine doses is the increase in the expressions of *BAK1*, *BAD*, and *BNIP3*. *BAK1* gene encodes the Bak protein, which participates in the formation of mitochondrial transmembrane channels and mediates the release of proapoptotic factors from the intra-mitochondrial space, including cytochrome c [22,23]. The active form of the BAD protein, encoded by the *BAD* gene, has the ability to form heterodimers with antiapoptotic Bcl-2 and Bcl-xL proteins, enhancing the proapoptotic activity of the BAX and Bak proteins [59]. The BNIP3 protein belongs to the group of BOP proteins (BH3-only proteins) belonging to the *BCL2* family. This protein is responsible for the neutralization of anti-apoptotic proteins after the onset of the mitochondrial permeabilizing membrane [60,61]. It can be concluded that the effect on the balance between the expression levels of pro- and antiapoptotic genes of the *BCL2* family is regulated by sanguinarine and *Chelidonium majus* L. extract via the BNIP3 protein. Sanguinarine seems to be the main factor inducing apoptosis of cells exposed to *Chelidonium majus* L., despite a lower amount of this compound compared to the amounts of other alkaloids in this plant [51,54].

It should be noted that in the cells exposed to the higher doses of sanguinarine, the expression of *BAK1*, *BAD*, and *BNIP3* was lower, suggesting the lower proapoptotic potential of sanguinarine in higher doses.

Both the PARAFAC analysis and the clustering with the Euclidean distances indicated that CCFR/CEM cell line clearly differed from other cell lines after exposure to all doses of sanguinarine. In this cell line, all doses of sanguinarine increased the expression of almost all analyzed genes. This is particularly important in the case of the *TP53* gene, whose expression was strongly elevated in the CCRF/CEM cells (for IC50 by 303% and for IC90 by 342%), but in other cell lines after exposure to the IC50 and IC90 doses of sanguinarine was reduced.

PARAFAC analysis and Euclidean clustering showed that the HL60 line is an outlier after exposure to all doses of *Chelidonium majus* L. extract. In these cells, as well as in the HL-60/MX1 cells, the anti-apoptotic *BCL2* expression was lowered. The remaining cell lines responded with an increase in *BCL2* gene expression after exposure to all doses of *Chelidonium majus* L. extract. This result indicates that the sensitivity of HL60 and HL60/MX1 cells to the proapoptotic action of *Chelidonium majus* L. extract is mediated by the downregulation of *BCL2*.

In this research, we evaluated the effect of berberine and *Berberis thunbergii* DC. extract for the transcription of 18 apoptosis-related genes in hematopoietic cancer cell lines. Berberine is a particularly interesting compound because it is characterized by a lack of toxicity in relation to normal cells, while the cytotoxicity towards cancer cells were reported [30]. Despite the proven proapoptotic activity of the compound [31,32], the role of individual genes in the regulation of programmed cell death exposed to berberine has been poorly investigated.

In the current study, a high increase in *CASP3* expression was observed in the CEM/C1, CCRF/CEM, and HL-60/MX2 cells after exposure to all doses of berberine. This result clearly indicates that the induction of cell death of these lines was in the apoptosis way. Moreover, an increase in *CASP9* expression, observed in the case of all cell lines exposed to berberine in the IC10, IC50, and IC90 doses, suggests the induction of programmed cell death with the intrinsic pathway.

The multidrug resistant HL-60/MX2 cell line was the most sensitivity to sanguinarine and was characterized by higher upregulation of proapoptotic genes compared to HL-60 and HL-60/MX1 cells, which exhibit weaker multidrug resistance potential (Table 1, Table 2). It may suggest that sanguinarine has an ability to break the multidrug resistance and induce apoptosis in HL-60/MX2 cells and the mechanisms determining the resistance may facilitate the cytotoxic effect of this alkaloid. This hypothesis is an interesting path for further investigations. 

Euclidean clustering and the PARAFAC analysis showed that the cells of the HL-60/MX2 line exposed to berberine clearly differ from the other examined cell lines. HL-60/MX2 cell line differs in the DNA profile from the HL-60/MX1 clone by the presence of the 11 allele in the TPOX locus. This increases the multidrug resistance of the HL-60/MX2 cells, which may be responsible for the high resistance to the cytotoxic action of berberine and *Chelidonium majus* L. extract (Table 1). Despite low cytotoxicity, analysis of changes in gene expression in HL-60/MX2 cells exposed to berberine showed the greatest modulation among all tested samples. Berberine caused a significant increase in the expression of caspases *CASP3*, *CASP8*, and *CASP9*, as well as proapoptotic genes *BAX*, *BAK1*, and *BIK*, accompanied by downregulation of the anti-apoptotic *BCL2* and *BCL2L2*, as well as *BNIP1*, *BNIP3*, and *BNIP3*. It follows that gene regulation is moving towards the induction of apoptosis; however, this process is clearly slower than in the case of sanguinarine. For a more in-depth analysis of berberine-induced apoptosis, further experiments in the 72- and 96-h model should be carried out.

Exposure to the *Berberis thunbergii* DC. extract increased the expression of *BAX*, *BAK1*, *BIK*, and *CASP9* in the examined cell lines, however, was weaker when compared to berberine. This result indicates the possibility of inducing the internal pathway of apoptosis, however, similarly to berberine, signs of apoptosis could possibly be detected after exposure longer than 24 h. The berberine content in the tested dose of extract was lower than the corresponding doses of berberine (IC10, IC50, IC90), which may explain a lower increase in the expression of the same genes. It may also suggest the possibility of proapoptotic activity of other compounds present in the extract, whose identification requires further investigation. Our results confirmed that berberine and the *Berberis thunbergii* DC. extract have an activity promoting the apoptosis with minimal cytotoxicity in studied concentrations. 

The limitation of this study is assessment of cytotoxicity and gene expression in exposed cells independently from cell population growth parameters, like doubling time or maximum growth rate. Gene expression was evaluated after 24 h of exposure, however, mRNA levels may change in shorter time (couple of hours, even minutes or seconds) and be a result of complex interaction with other RNAs. The influx and efflux time in relation to concentration of study samples could also affect the outcome. The influence of these variables on the sensitivity of the cells to cytotoxic activity of studied compounds should be elucidated in further investigations with optimized time points of the exposure.

Observations presented in current study may have a significant impact on the understanding of the regulation of apoptosis at the transcriptome level after exposure to sanguinarine, berberine and extracts from *Chelidonium majus* L. and *Berberis thunbergii* DC. Understanding the mechanisms that accompany the change in the expression of the studied genes is substantial when trying to determine the effects associated with the interaction of sanguinarine, berberine and the extracts studied for their anticancer activity. The obtained results significantly enrich the knowledge of the antineoplastic activity of substances and may accelerate their introduction into the clinical trial phase in the treatment of various types of leukemia.

## 4. Materials and Methods 

### 4.1. Sanguinarine and Berberine Stock Solutions

The reference compounds sanguinarine and berberine were of analytical grade from Sigma-Aldrich Company (St. Louis, MO, USA). The purity of each compound was more than 98%, according to the manufacturer. 10 mM stock solution of sanguinarine in DMSO was prepared. For the cytotoxic study, a series of dilutions was prepared with concentrations 0.8, 0.6, 0.5, 0.4, 0.2, 0.1 and 0.01 mM. The final concentration of sanguinarine in cell suspensions were 1000-fold lower. Due to the limited solubility of berberine in DMSO (maximum 50 mM), berberine dilutions were prepared in 25 mM, 10 mM and 5 mM concentrations.

### 4.2. Plant Extracts Preparation

Plant material selection and procedure of extraction were carried out as previously described [51]. For cytotoxicity tests, 1 mL of methanolic extracts were evaporated in a nitrous atmosphere and resolved in 1 mL DMSO, obtaining 56 mg/mL concentration of *Chelidonium majus* L. extract and 29.1 mg/mL concentration of *Berberis thunbergii* DC. extract. Due to a harmful effect of DMSO to cells [62], to the cell suspension was added no more than 5 µL of sample per 1000 µL of cell suspension.

### 4.3. Cell Lines

HL-60 (CCL-240), HL-60/MX1 (CRL-2258), HL-60/MX2 (CRL-2257), CCRF/CEM (CCL-119) and CEM/C1 (CRL-2265) cell lines were from ATCC collection and were purchased from LGC Standards, UK. J45.01 (ECACC 93031145-1VL) and U266B1 (ECACC 85051003-1VL) cell lines were from ECACC collection and were purchased from Sigma-Aldrich Co. (St. Louis, MO, USA). Before experiments, 1 mL of cell suspension, containing 5–9 × 105 cells, was cultured in sterile 12-wells plates (3.8 cm^2^ per well) in standard conditions (5% CO_2_, 37 °C) for 24 h using Galaxy B incubator (RS Biotech, Irvine, UK).

### 4.4. Trypan Blue Staining

Sanguinarine and berberine stock solutions and examined extracts were added to cell cultures after 24 h incubation. After 24 h of exposure to studied samples, cell suspensions were centrifuged at 800 rpm for 10 min (Eppendorf 5810R centrifuge, Eppendorf AG., Hamburg, Germany). The supernatant was discarded, to the cells were added 1 mL PBS (Phosphate Buffered Saline, Biomed-Lublin WSiS, Lublin, Poland) and centrifugation was repeated. The supernatant was removed and the cells were resuspended in 50 µL of PBS. Subsequently, 10 µL of suspension was mixed with 10 µL of trypan blue solution (0.4% trypan blue in 0.81% sodium chloride and 0.06% potassium dihydrogen phosphate, Bio-Rad, Hercules, CA, USA) and incubated for 1 min in 37 °C. The total number of cells, as well as the percentages of normal and death cells, were counted on Counting Slides using Automated Cell Counter (Bio-Rad). The experiments were performed in triplicates on independent cell lines passages and the mean values were calculated. The curves presenting relationships between cell viability and the concentration of the studied samples were prepared in order to determine IC10, IC50 and IC90 doses for each of the studied cell lines.

### 4.5. RNA Isolation

After 24 h exposure to IC10, IC50 and IC90 doses of studied samples, cells were centrifuged at 800 rpm for 10 min and the supernatant was discarded. Total RNA was isolated from the cells using Chomczyński and Sacchi method [63] and TRI reagent (Sigma-Aldrich Co., St. Louis, MO, USA), according to the manufacturer procedure. Briefly, 500 µL of TRI reagent was added to the cells and the suspension was shaken for 10 min on IKA MS 3 Basic shaker (IKA WERKE Gmbh, Staufen, Germany). Subsequently, 50 µL of chloroform was added, the mixture was shaken for 10 min and centrifuged at 14,000 rpm for 15 min at 4 °C. The aqueous layer was collected to a new tube and 250 µL of isopropanol was added to the collected layer. The mixture was incubated for 15 min at room temperature and centrifuged (14,000 rpm, 4 °C, 10 min). The supernatant was discarded, RNA pellets were washed with 75% ethanol, dried and dissolved in 20 µL of RNAse-free water.

The quality and quantity of isolated RNA was assessed by NanoDrop2000 UV-VIS spectrophotometer with NanoDrop2000 Operating Software (Thermo Fisher Scientific Inc., Waltham, MA, USA). RNA samples with A260/A280 ratio ranged between 1.8 and 2.0 were intended to further investigations.

### 4.6. cDNA Synthesis

Synthesis of cDNA was performed using High Capacity cDNA Reverse Transcription Kit (Applied Biosystems, Foster City, CA, USA), according to the manufacturer procedure. Briefly, the following reaction mixture was assembled on ice: 2 µL of 10X RT Buffer, 0.8 µL of 25X dNTP Mix (100 mM), 2 µL of 10X RT Random Primers, 1 µL of MultiScribe Reverse Transcriptase (50 U/µL), 0.5 µL of RNase Inhibitor (40 U/µL), 1 µL of isolated RNA and 1 µL of DEPC-treated nuclease-free water. The reaction mixture was incubated in thermocycler Mastercycler Personal (Eppendorf AG., Hamburg, Germany) in the following conditions: 25 °C for 10 min, 37 °C for 120 min and 85 °C for 5 s. Obtained cDNA samples were stored in −20 °C. 

### 4.7. Real-Time PCR

Gene expression analysis was carried out for 18 genes related to apoptosis (*B2M*, *BAD*, *BAK1*, *BAX*, *BCL2*, *BCL2L1*, *BCL2L2*, *BID*, *BIK*, *BNIP1*, *BNIP2*, *BNIP3*, *CASP3*, *CASP8*, *CASP9*, *MCL1*, *PMAIP1*, and *TP53*) using real-time PCR method. cDNA samples were amplified using a 7900HT Fast Real-Time PCR System (Applied Biosystems, Foster City, CA, USA). Reaction mixtures contained 1.25 µL of gene-specific TaqMan probe (Applied Biosystems, USA) described in Table A1, 12.5 µL of TaqMan Gene Expression Master Mix (Applied Biosystems, USA), and 11.25 µL of cDNA sample. PCR reactions were performed in µAmp Optical 96-Well Reaction Plates (Life Technologies Corporation, Carlsbad, CA, USA). The expression of GAPDH was used as an endogenous control. The reaction was conducted in the following conditions: 95 °C for 10 min, 40 cycles: 95 °C for 15 s, and 60 °C for 60 s. Gene expression levels (Ct) obtained in exposed cells were compared to the expression levels in no exposed cells (control) using the relative quantitation method (RQ = 2^−ΔΔCt^) [64,65]. The experiments were performed in quadruplicate and the mean values were calculated. Data was analyzed using SDS 2.4 Study software (Applied Biosystems, USA).

### 4.8. Statistical Analysis

Clustering analysis with Euclidean distance and parallel factor analysis (PARAFAC) were applied to reduce the large dimensionality of the data and to assess the similarities between the doses of the tested compounds, between the analyzed cell lines, and between changes in the expression of examined genes after exposure to the samples. The analysis was performed using R programming software. The analysis did not include data obtained after exposure to the *Berberis thunbergii* DC. extract due to the lack of cytotoxicity in relation to the tested cell lines.

## Figures and Tables

**Table 1:** IC10, IC50, and IC90 inhibitory concentrations determined for sanguinarine, berberine, and *Chelidonium majus L.* extract toward seven tested hematopoietic cell lines. Note: SD = standard deviation, * = maximum concentration of exposure.

Cell Line	IC10	IC50	IC90
Sanguinarine (µM ± SD)			
J45.01	0.10 ± 0.04	0.50 ± 0.04	1.00 ± 0.05
U266B1	0.80 ± 0.04	1.05 ± 0.05	1.80 ± 0.04
HL-60	0.20 ± 0.03	0.60 ± 0.06	1.80 ± 0.03
HL-60/MX1	0.15 ± 0.03	0.50 ± 0.04	1.80 ± 0.05
HL-60/MX2	0.06 ± 0.05	0.10 ± 0.05	1.20 ± 0.05
CCRF/CEM	0.50 ± 0.04	0.70 ± 0.03	1.20 ± 0.03
CEM/C1	0.30 ± 0.04	0.50 ± 0.04	1.00 ± 0.03
Berberine (µM ± SD)			
J45.01	25.15 ± 3.15	80.15 ± 4.65	200.80 ± 4.65
U266B1	125.15 ± 2.68	240.45 ± 4.15	250.00 ± 1.10*
HL-60	50.32 ± 4.56	90.45 ± 5.83	250.00 ± 4.35*
HL-60/MX1	25.05 ± 2.13	110.05 ± 6.72	250.00 ± 3.15*
HL-60/MX2	75.25 ± 6.52	250.00 ± 2.15*	-
CCRF/CEM	50.40 ± 1.18	80.00 ± 2.13	130.25 ± 1.18
CEM/C1	50.25 ± 4.25	225.15 ± 5.25	250.00 ± 2.85*
*Chelidonium majus* L. extract (µg/mL ± SD)			
J45.01	5.05 ± 2.15	12.25 ± 2.85	38.65 ± 5.23
U266B1	8.05 ± 3.45	21.50 ± 5.65	264.50 ± 4.12
HL-60	9.01 ± 2.35	13.82 ± 3.15	280.02 ± 6.15
HL-60/MX1	7.81 ± 6.18	20.15 ± 4.16	202.11 ± 4.32
HL-60/MX2	19.85 ± 5.68	64.50 ± 5.48	236.0 ± 4.82
CCRF/CEM	7.58 ± 2.89	10.75 ± 2.15	27.75 ± 1.63
CEM/C1	7.33 ± 5.48	13.25 ± 3.23	27.30 ± 1.89

**Table 2:** Differentially expressed genes associated with apoptosis in studied hematopoietic cell lines exposed to IC10, IC50, and IC90 doses of sanguinarine, berberine, and *Chelidonium majus* L. extract, as well as to *Berberis thunbergii* DC. extract at a concentration of 145.5 µg/mL. Table presents upregulated genes with logRQ > 0.5 and downregulated genes with logRQ < −0.5. The lack of genes meeting the assumed criteria was marked with “NA”.

Cell Line	IC10	IC50	IC90
Sanguinarine			
J45.01	↑(*BNIP3*), ↓(*BCL2L2*, *TP53*)	↑(*BCL2*, *BNIP3*), ↓(*BAD*, *TP53*)	↑(*BCL2*, *BNIP3*), ↓(*TP53*)
U266B1	↑(*BAK1*, *BNIP3*, *CASP8*, *MCL1*, *PMAIP1*)	↑(*BAK1*, *BCL2*, *BNIP3*)	↑(*BNIP3*)
HL-60	↑(*PMAIP1*, *TP53*)	↑(*BAD*)	NA
HL-60/MX1	↑(*BAK1*, *CASP8*, *PMAIP1*), ↓(*BCL2L2*, *BID*)	↑(*BCL2*), ↓(*TP53*)	↓(*BCL2L2*, *BNIP2*, *CASP3*, *TP53*)
HL-60/MX2	↑(*BAK1*, *BAX*, *BIK*, *CASP3*, *CASP8*, *MCL1*, *PMAIP1*, *TP53*)	↓(*BAD*, *BNIP2*, *PMAIP1*, *TP53*)	↓(*BAD*, *BNIP2*, *TP53*)
CCRF/CEM	(*B2M*, *BAD*, *BAK1*, *BAX*, *BCL2*, *BCL2L1*, *BCL2L2*, *BID*, *BIK*, *BNIP1*, *BNIP2*, *BNIP3*, *CASP3*, *CASP8*, *CASP9*, *MCL1*, *PMAIP1*, *TP53*)	↑(*BAK1*, *BNIP3*, *CASP8*, *PMAIP1*, *TP53*)	↑(*BAD*, *BAK1*, *BCL2*, *BNIP2*, *BNIP3*, *CASP8*, *CASP9*, *MCL1*, *PMAIP1*, *TP53*)
CEM/C1	↑(*BAD*, *BAK1*, *BAX*, *BCL2*, *BCL2L2*, *BNIP1*, *BNIP2*, *CASP3*, *CASP8*, *MCL1*, *PMAIP1*, *TP53*)	↑(*BAK1*, *MCL1*)	↓(*BNIP3*)
Berberine			
J45.01	↑(*BAK1*, *BCL2*, *BCL2L2*, *BID*, *BNIP3*, *CASP9*)	↓(*BCL2L2*)	↑(*BNIP3*, *CASP9*)
U266B1	NA	↑(*BAK1*, *BNIP3*, *CASP9*), ↓(*BCL2L2*, *BNIP2*, *MCL1*, *TP53*)	↑(*BAK1*, *BAX*, *BNIP3*, *CASP9*), ↓(*BCL2*, *BIK*, *BNIP2*)
HL-60	↓(*BAK1*, *BNIP2*, *TP53*)	↓(*BAK1*, *BAX*, *BNIP2*, *BNIP3*, *TP53*)	↓(*TP53*)
HL-60/MX1	↑(*BAD*, *BAK1*, *BCL2*, *BNIP3*, *CASP9*, *TP53*), ↓(*BNIP2*, *MCL1*)	↑(*BCL2*), ↓(*BAK1*, *BNIP2*, *BNIP3*, *TP53*)	↑(*BAK1*, *BCL2*, *BNIP3*, *CASP9*, *PMAIP1*), ↓(*BNIP2*, *CASP8*, *MCL1*)
HL-60/MX2	↑(*BAD*, *BAK1*, *BAX*, *BCL2*, *BCL2L1*, *BCL2L2*, *BID*, *BIK*, *BNIP1*, *BNIP2*, *BNIP3*, *CASP3*, *CASP8*, *CASP9*, *MCL1*, *PMAIP1*, *TP53*)	↑(*BAX*, *BIK*, *CASP3*, *CASP9*, *MCL1*, *TP53*)	NA
CCRF/CEM	↑(*TP53*), ↓(*BAD*, *BAK1*, *BNIP2*)	↑(*TP53*), ↓(*BAD*, *BAK1*)	↑(*TP53*), ↓(*BAD*, *BAK1*)
CEM/C1	NA	NA	↑(*B2M*, *BAK1*, *BAX*, *BCL2*, *BNIP3*, *CASP9*, *PMAIP1*), ↓(*BNIP2*)
*Chelidonium majus* L. extract			
J45.01	↑(*BAK1*, *BCL2*, *BNIP3*, *CASP8*, *CASP9*, *MCL1*, *PMAIP1*)	↑(*BNIP3*), ↓(*BAD*, *BCL2L2*, *TP53*)	↑(*BNIP3*), ↓(*TP53*)
U266B1	↑(*BAK1*)	↑(*BAK1*, *BCL2*, *BCL2L2*, *BNIP2*)	↑(*BAK1*)
HL-60	↑(*BAK1*, *BCL2L2*, *MCL1*)	↑(*BAK1*)	↓(*TP53*)
HL-60/MX1	↑(*BAK1*, *BCL2L2*, *MCL1*, *TP53*)	↑(*BAK1*, *TP53*)	↑(*TP53*)
HL-60/MX2	↑(*BAK1*, *BAX*, *MCL1*), ↓(*TP53*)	↑(*BAK1*, *BAX*, *CASP3*, *MCL1*), ↓(*TP53*)	↑(*BAK1*, *BAX*, *MCL1*)
CCRF/CEM	↑(*BCL2*, *BCL2L2*, *BNIP2*, *BNIP3*, *CASP8*, *MCL1*, *PMAIP1*, *TP53*)	↑(*BAK1*, *BCL2L2*, *BIK*, *BNIP2*, *TP53*)	↑(*TP53*), ↓(*BAD*)
CEM/C1	↑(*BAK1*, *BCL2L2*, *CASP8*, *MCL1*, *PMAIP3*)	↑(*BAK1*)	↑(*BAD*, *BAK1*, *BCL2L1*, *BCL2L2*, *BNIP2*)
*Berberis thunbergii* DC. extract (145.5 µg/mL)			
J45.01	↑(*BAK1*, *BAX*, *BCL2*, *BID*, *BNIP1*, *BNIP3*, *CASP9*), ↓(*BNIP2*, *TP53*)		
U266B1	↓(*BNIP3*)		
HL-60	↑(*BAK1*, *BNIP3*, *CASP9*, *TP53*), ↓(*BCL2L1*, *BIK*, *BNIP2*, *CASP3*, *CASP8*, MCLI)		
HL-60/MX1	↑(*BCL2*), ↓(*BNIP2*, *TP53*)		
HL-60/MX2	↑(*BAX*, *BIK*, *CASP3*, *MCL1*), ↓(*TP53*)		
CCRF/CEM	NA		
CEM/C1	↑(*BCL2*), ↓(*BAD*, *BAK1*, *TP53*)		

## Supplementary Materials

The following are available online at https://www.mdpi.com/2072-6651/11/9/485/s1, Figure S1: Expression profiles of 18 apoptosis-related genes in U266B1 cell line exposed to IC10 (upper panel), IC50 (middle panel) and IC90 (lower panel) of sanguinarine (blue), berberine (yellow) and *Chelidonium majus* L. extract (red), Figure S2: Expression profiles of 18 apoptosis-related genes in CEM/C1 cell line exposed to IC10 (upper panel), IC50 (middle panel) and IC90 (lower panel) of sanguinarine (blue), berberine (yellow) and *Chelidonium majus* L. extract (red), Figure S3: Expression profiles of 18 apoptosis-related genes in CCRF/CEM cell line exposed to IC10 (upper panel), IC50 (middle panel) and IC90 (lower panel) of sanguinarine (blue), berberine (yellow) and *Chelidonium majus* L. extract (red), Figure S4: Expression profiles of 18 apoptosis-related genes in HL60 cell line exposed to IC10 (upper panel), IC50 (middle panel) and IC90 (lower panel) of sanguinarine (blue), berberine (yellow) and *Chelidonium majus* L. extract (red), Figure S5: Expression profiles of 18 apoptosis-related genes in HL60/MX1 cell line exposed to IC10 (upper panel), IC50 (middle panel) and IC90 (lower panel) of sanguinarine (blue), berberine (yellow) and *Chelidonium majus* L. extract (red), Figure S6: Expression profiles of 18 apoptosis-related genes in HL60/MX2 cell line exposed to IC10 (upper panel), IC50 (middle panel) and IC90 (lower panel) of sanguinarine (blue), berberine (yellow) and *Chelidonium majus* L. extract (red), Figure S7: Expression profiles of 18 apoptosis-related genes in J45.01 cell line exposed to IC10 (upper panel), IC50 (middle panel) and IC90 (lower panel) of sanguinarine (blue), berberine (yellow) and *Chelidonium majus* L. extract (red), Figure S8: Expression profiles of 18 apoptosis-related genes in seven hematopoietic cell lines exposed to *Berberis thunbergii* DC. extract at a concentration of 145.5 µg/mL, Table S1: logRQ values for technical replicates, means and Standard Deviations calculated for changes in expression of 18 genes related to apoptosis (*B2M*, *BAD*, *BAK1*, *BAX*, *BCL2*, *BCL2L1*, *BCL2L2*, *BID*, *BIK*, *BNIP1*, *BNIP2*, *BNIP3*, *CASP3*, *CASP8*, *CASP9*, *MCL1*, *PMAIP1*, *TP53*) in seven hematopoietic cancer cell lines (CEM/C1, J45.01, CCRF/CEM, HL-60, HL-60/MX1, HL-60/MX2 and U266B1) after 24 h exposure to IC10 concentration of berberine, Table S2: logRQ values for technical replicates, means and Standard Deviations calculated for changes in expression of 18 genes related to apoptosis *(B2M*, *BAD*, *BAK1*, *BAX*, *BCL2*, *BCL2L1*, *BCL2L2*, *BID*, *BIK*, *BNIP1*, *BNIP2*, *BNIP3*, *CASP3*, *CASP8*, *CASP9*, *MCL1*, *PMAIP1*, *TP53*) in seven hematopoietic cancer cell lines (CEM/C1, J45.01, CCRF/CEM, HL-60, HL-60/MX1, HL-60/MX2 and U266B1) after 24 h exposure to IC50 concentration of berberine, Table S3: logRQ values for technical replicates, means and Standard Deviations calculated for changes in expression of 18 genes related to apoptosis (*B2M*, *BAD*, *BAK1*, *BAX*, *BCL2*, *BCL2L1*, *BCL2L2*, *BID*, *BIK*, *BNIP1*, *BNIP2*, *BNIP3*, *CASP3*, *CASP8*, *CASP9*, *MCL1*, *PMAIP1*, *TP53*) in seven hematopoietic cancer cell lines (CEM/C1, J45.01, CCRF/CEM, HL-60, HL-60/MX1 and U266B1) after 24 h exposure to IC90 concentration of berberine, Table S4: logRQ values for technical replicates, means and Standard Deviations calculated for changes in expression of 18 genes related to apoptosis (*B2M*, *BAD*, *BAK1*, *BAX*, *BCL2*, *BCL2L1*, *BCL2L2*, *BID*, *BIK*, *BNIP1*, *BNIP2*, *BNIP3*, *CASP3*, *CASP8*, *CASP9*, *MCL1*, *PMAIP1*, *TP53*) in seven hematopoietic cancer cell lines (CEM/C1, J45.01, CCRF/CEM, HL-60, HL-60/MX1, HL-60/MX2 and U266B1) after 24 h exposure to IC10 concentration of Chelidonium majus L. extract, Table S5: logRQ values for technical replicates, means and Standard Deviations calculated for changes in expression of 18 genes related to apoptosis (*B2M*, *BAD*, *BAK1*, *BAX*, *BCL2*, *BCL2L1*, *BCL2L2*, *BID*, *BIK*, *BNIP1*, *BNIP2*, *BNIP3*, *CASP3*, *CASP8*, *CASP9*, *MCL1*, *PMAIP1*, *TP53*) in seven hematopoietic cancer cell lines (CEM/C1, J45.01, CCRF/CEM, HL-60, HL-60/MX1, HL-60/MX2 and U266B1) after 24 h exposure to IC50 concentration of Chelidonium majus L. extract, Table S6: logRQ values for technical replicates, means and Standard Deviations calculated for changes in expression of 18 genes related to apoptosis (*B2M*, *BAD*, *BAK1*, *BAX*, *BCL2*, *BCL2L1*, *BCL2L2*, *BID*, *BIK*, *BNIP1*, *BNIP2*, *BNIP3*, *CASP3*, *CASP8*, *CASP9*, *MCL1*, *PMAIP1*, *TP53*) in seven hematopoietic cancer cell lines (CEM/C1, J45.01, CCRF/CEM, HL-60, HL-60/MX1, HL-60/MX2 and U266B1) after 24 h exposure to IC90 concentration of Chelidonium majus L. extract, Table S7: logRQ values for technical replicates, means and Standard Deviations calculated for changes in expression of 18 genes related to apoptosis (*B2M*, *BAD*, *BAK1*, *BAX*, *BCL2*, *BCL2L1*, *BCL2L2*, *BID*, *BIK*, *BNIP1*, *BNIP2*, *BNIP3*, *CASP3*, *CASP8*, *CASP9*, *MCL1*, *PMAIP1*, *TP53*) in seven hematopoietic cancer cell lines (CEM/C1, J45.01, CCRF/CEM, HL-60, HL-60/MX1, HL-60/MX2 and U266B1) after 24 h exposure to IC10 concentration of sanguinarine, Table S8: logRQ values for technical replicates, means and Standard Deviations calculated for changes in expression of 18 genes related to apoptosis (*B2M*, *BAD*, *BAK1*, *BAX*, *BCL2*, *BCL2L1*, *BCL2L2*, *BID*, *BIK*, *BNIP1*, *BNIP2*, *BNIP3*, *CASP3*, *CASP8*, *CASP9*, *MCL1*, *PMAIP1*, *TP53*) in seven hematopoietic cancer cell lines (CEM/C1, J45.01, CCRF/CEM, HL-60, HL-60/MX1, HL-60/MX2 and U266B1) after 24 h exposure to IC50 concentration of sanguinarine, Table S9: logRQ values for technical replicates, means and Standard Deviations calculated for changes in expression of 18 genes related to apoptosis (*B2M*, *BAD*, *BAK1*, *BAX*, *BCL2*, *BCL2L1*, *BCL2L2*, *BID*, *BIK*, *BNIP1*, *BNIP2*, *BNIP3*, *CASP3*, *CASP8*, *CASP9*, *MCL1*, *PMAIP1*, *TP53*) in seven hematopoietic cancer cell lines (CEM/C1, J45.01, CCRF/CEM, HL-60, HL-60/MX1, HL-60/MX2 and U266B1) after 24 h exposure to IC90 concentration of sanguinarine, Table S10: Mean values of logRQ calculated for changes in expression of 18 genes related to apoptosis (*B2M*, *BAD*, *BAK1*, *BAX*, *BCL2*, *BCL2L1*, *BCL2L2*, *BID*, *BIK*, *BNIP1*, *BNIP2*, *BNIP3*, *CASP3*, *CASP8*, *CASP9*, *MCL1*, *PMAIP1*, *TP53*) in seven hematopoietic cancer cell lines (CEM/C1, J45.01, CCRF/CEM, HL-60, HL-60/MX1, HL-60/MX2 and U266B1) after 24 h exposure to Berberis thunbergii DC. extract in concentration of 145.5 µg/mL.

## Appendix A

**Table A1:** The list of TaqMan probes (Applied Biosystems, Foster City, CA, USA) applied to the study.

Gene Symbol	Probe	Gene Name
*BAK1*	Hs00940249_m1	*BCL2* antagonist/killer 1
*BAX*	Hs00180363_m1	*BCL2* associated X, apoptosis regulator
*BCL2*	Hs00608023_m1	*BCL2* apoptosis regulator
*MCL1*	Hs01050896_m1	*MCL1* apoptosis regulator, *BCL2* family member
*BCL2L1*	Hs00236329_m1	*BCL2* like 1
*BCL2L2*	Hs00187848_m1	*BCL2* like 2
*BID*	Hs01026792_m1	*BH3* interacting domain death agonist
*BIK*	Hs00154189_m1	*BCL2* interacting killer
*BNIP1*	Hs00241824_m1	*BCL2* interacting protein 1
*BNIP2*	Hs00188939_m1	*BCL2* interacting protein 2
*BNIP3*	Hs00969291_m1	*BCL2* interacting protein 3
*PMAIP1*	Hs00560402_m1	Phorbol-12-myristate-13-acetate-induced protein 1
*BAD*	Hs00188930_m1	*BCL2* associated agonist of cell death
*CASP3*	Hs00234387_m1	Caspase 3
*CASP8*	Hs01018151_m1	Caspase 8
*CASP9*	Hs00154261_m1	Caspase 9
*TP53*	Hs01034249_m1	Tumor protein p53
*GAPDH* (Endogenous control)	Hs99999905_m1	Glyceraldehyde-3-phosphate dehydrogenase
