# Supplementary material for: Cytotoxic and Proapoptotic Activity of Sanguinarine, Berberine, and Extracts of Chelidonium majus L. and Berberis thunbergii DC. toward Hematopoietic Cancer Cell Lines
Source: Toxins (Basel). 2019 Aug 23;11(9):485. doi: 10.3390/toxins11090485 (PMC6784183; doi:10.3390/toxins11090485)
Supplement: Supplementary file 1 [file toxins-11-00485-s001.zip › Supplementary_Material_revised_v2_proof.pdf]

Supplementary Materials: Cytotoxic and Proapoptotic Activity of Sanguinarine, Berberine, and Extracts of *Chelidonium majus* L. and *Berberis thunbergii* DC. toward Hematopoietic Cancer Cell Lines

Anna Och, Daniel Zalewski, Łukasz Komsta, Przemysław Kołodziej, Janusz Kocki and Anna Bogucka-Kocka *

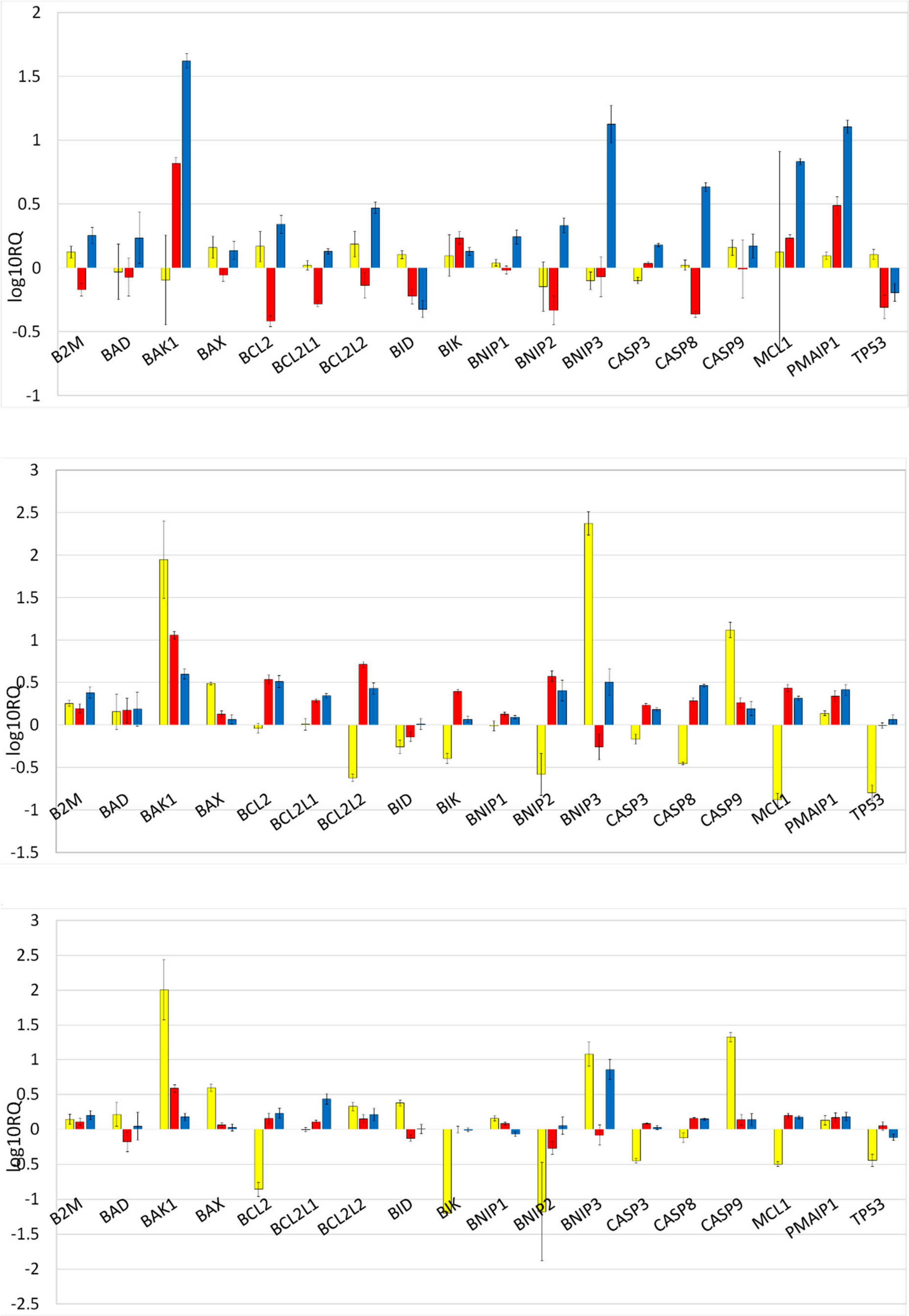

Figure S1. Expression profiles of 18 apoptosis-related genes in U266B1 cell line exposed to IC10 (upper panel), IC50 (middle panel) and IC90 (lower panel) of sanguinarine (blue), berberine (yellow) and *Chelidonium majus* L. extract (red).

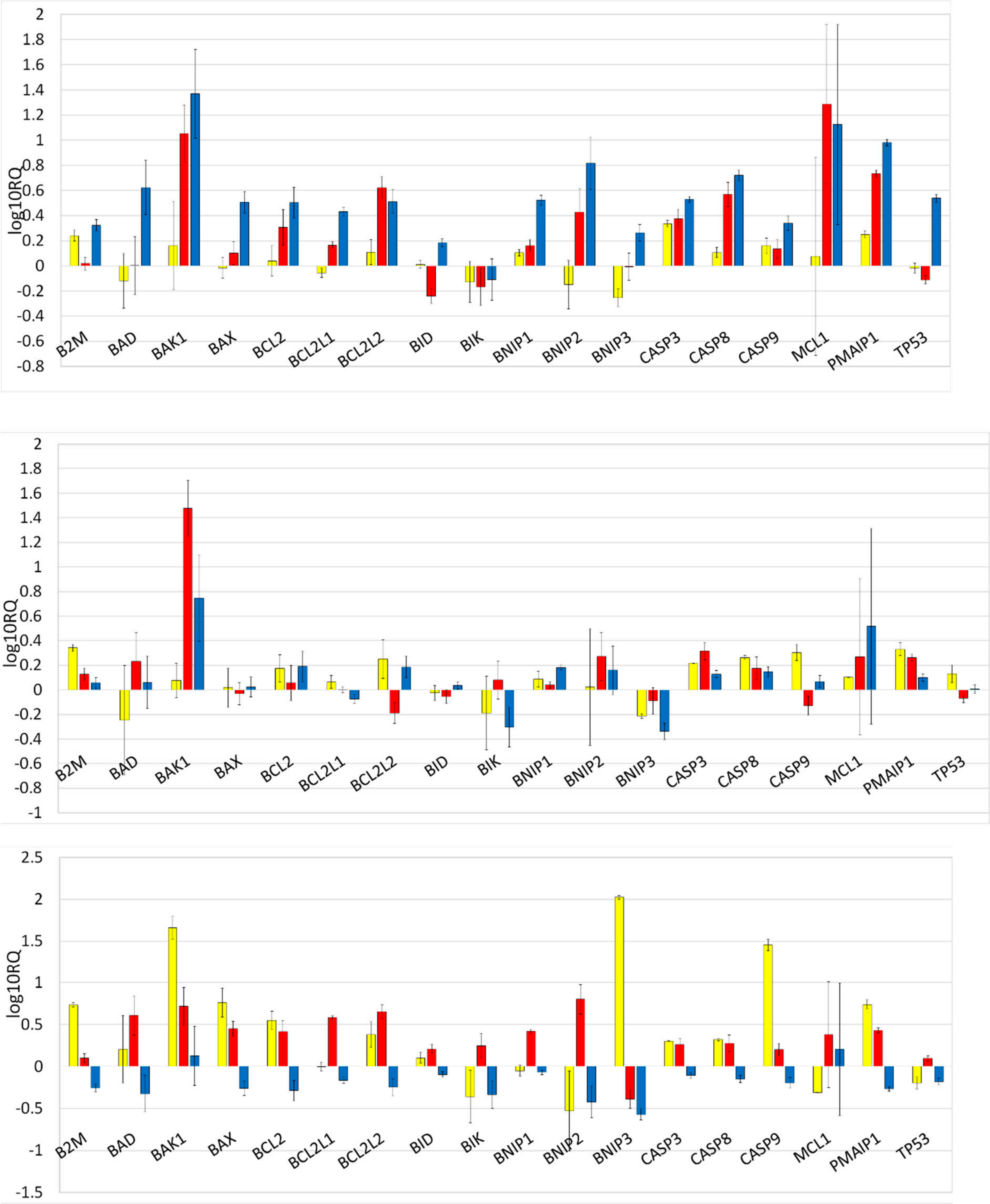

Figure S2. Expression profiles of 18 apoptosis-related genes in CEM/C1 cell line exposed to IC10 (upper panel), IC50 (middle panel) and IC90 (lower panel) of sanguinarine (blue), berberine (yellow) and *Chelidonium majus* L. extract (red).

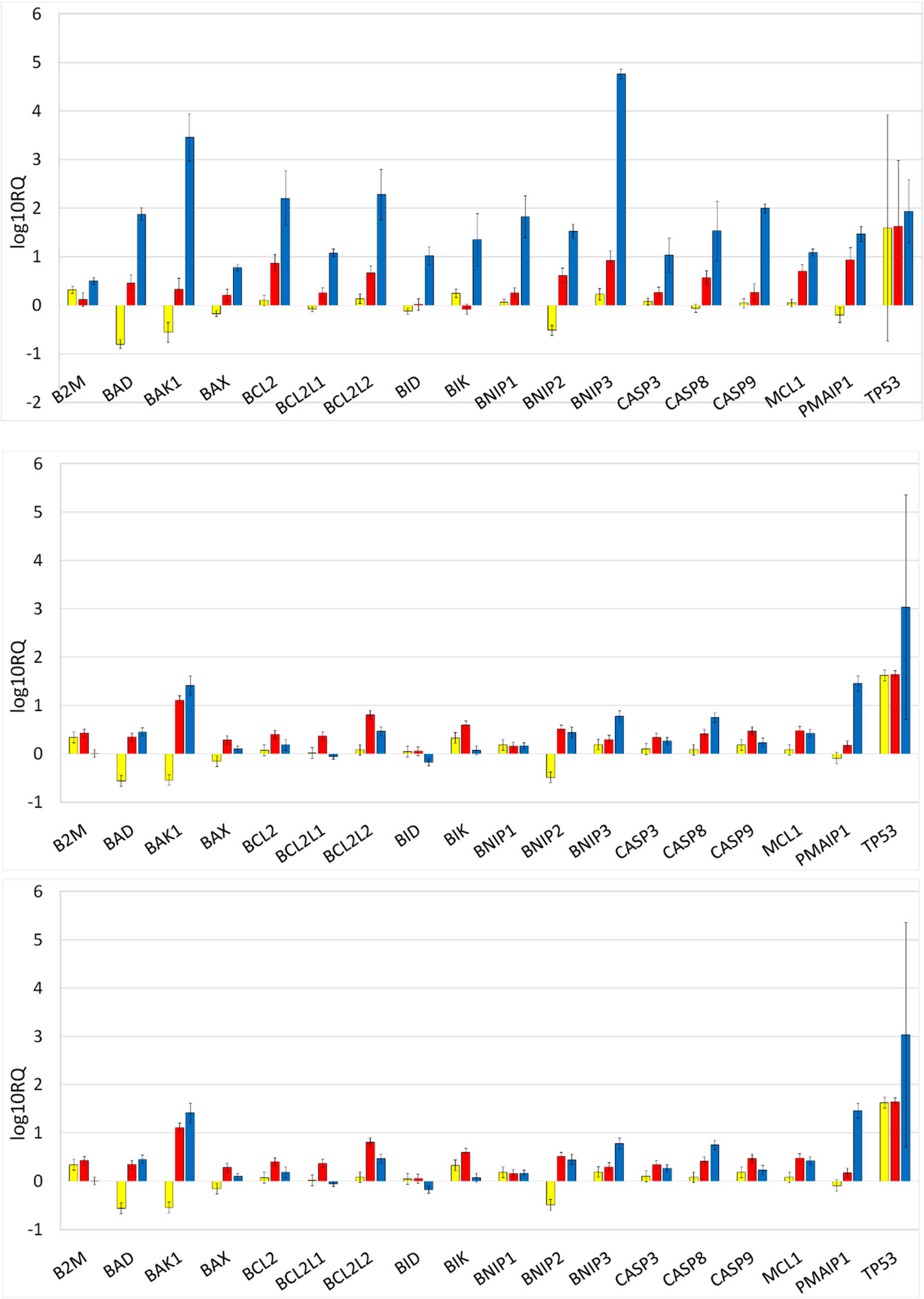

Figure S3. Expression profiles of 18 apoptosis-related genes in CCRF/CEM cell line exposed to IC10 (upper panel), IC50 (middle panel) and IC90 (lower panel) of sanguinarine (blue), berberine (yellow) and *Chelidonium majus* L. extract (red).

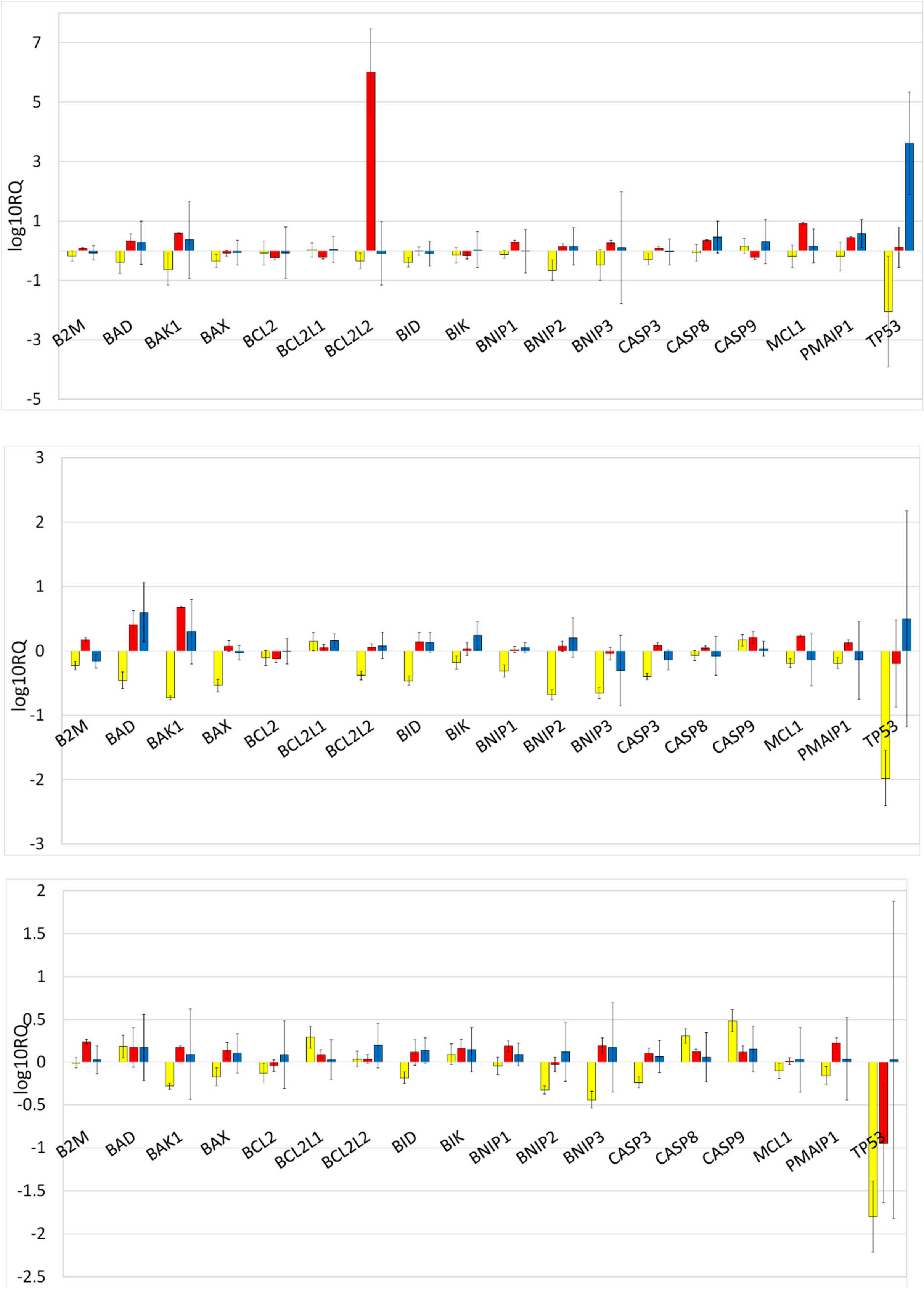

Figure S4. Expression profiles of 18 apoptosis-related genes in HL60 cell line exposed to IC10 (upper panel), IC50 (middle panel) and IC90 (lower panel) of sanguinarine (blue), berberine (yellow) and *Chelidonium majus* L. extract (red).

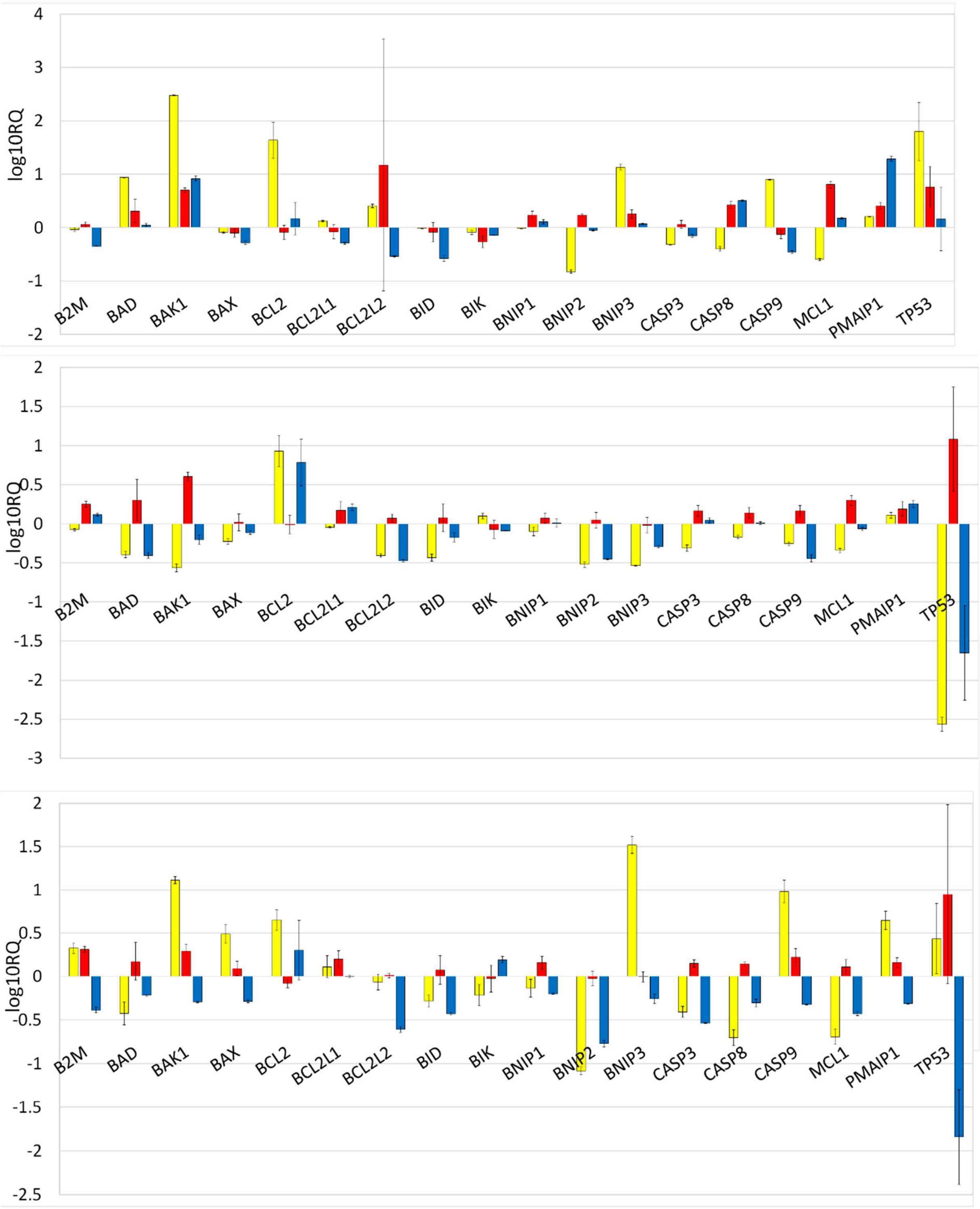

Figure S5. Expression profiles of 18 apoptosis-related genes in HL60/MX1 cell line exposed to IC10 (upper panel), IC50 (middle panel) and IC90 (lower panel) of sanguinarine (blue), berberine (yellow) and *Chelidonium majus* L. extract (red).

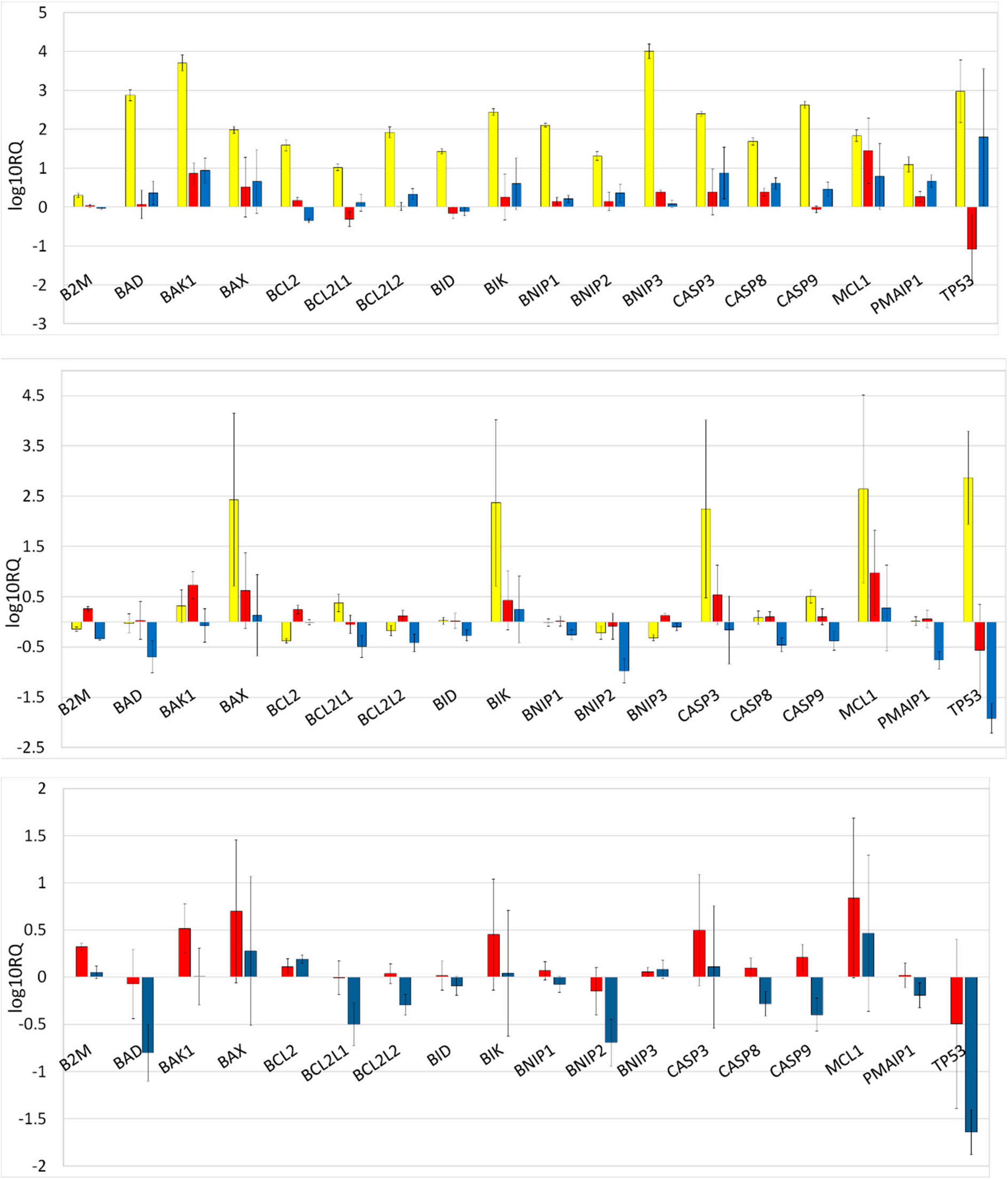

Figure S6. Expression profiles of 18 apoptosis-related genes in HL60/MX2 cell line exposed to IC10 (upper panel), IC50 (middle panel) and IC90 (lower panel) of sanguinarine (blue), berberine (yellow) and *Chelidonium majus* L. extract (red).

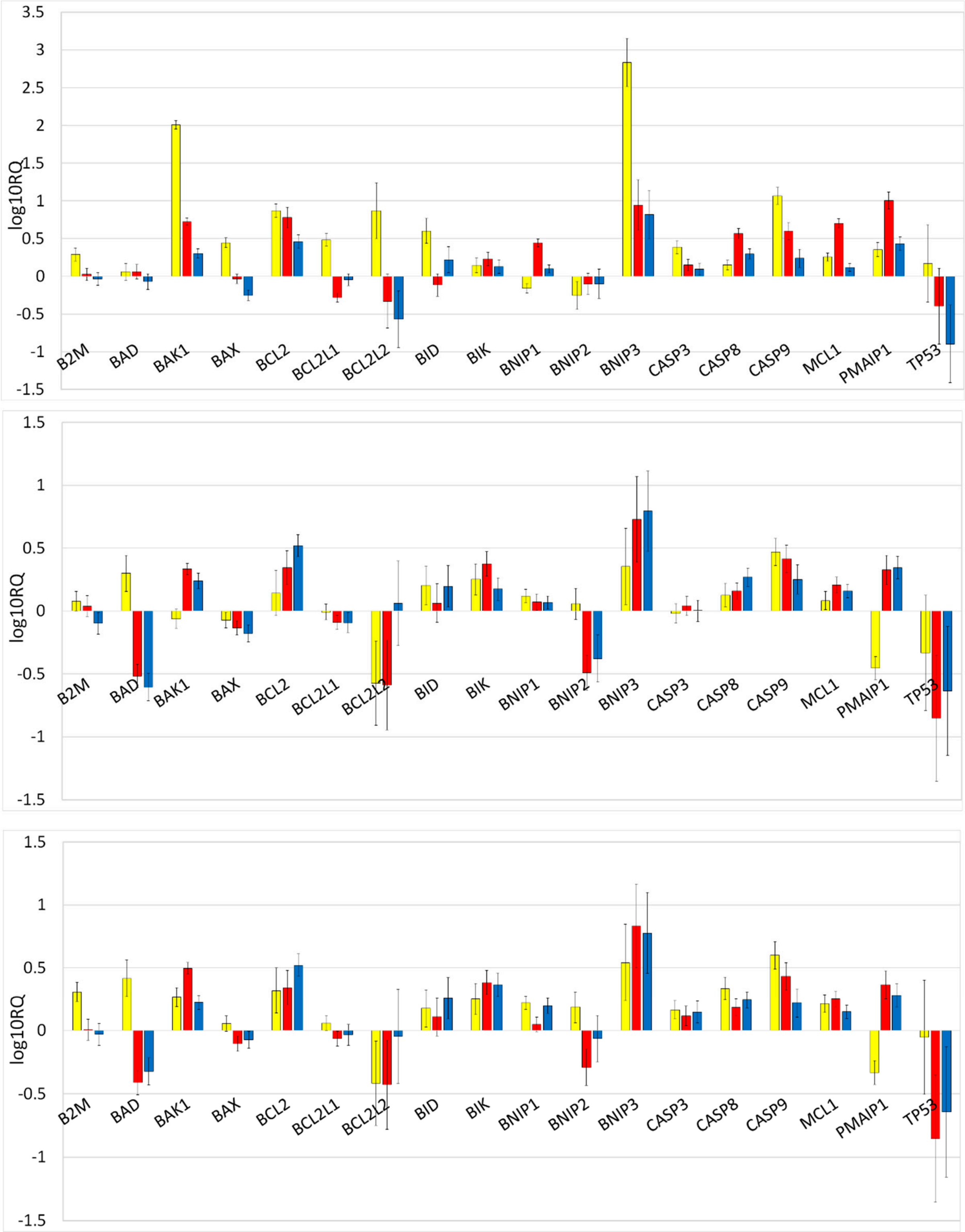

Figure S7. Expression profiles of 18 apoptosis-related genes in J45.01 cell line exposed to IC10 (upper panel), IC50 (middle panel) and IC90 (lower panel) of sanguinarine (blue), berberine (yellow) and *Chelidonium majus* L. extract (red).

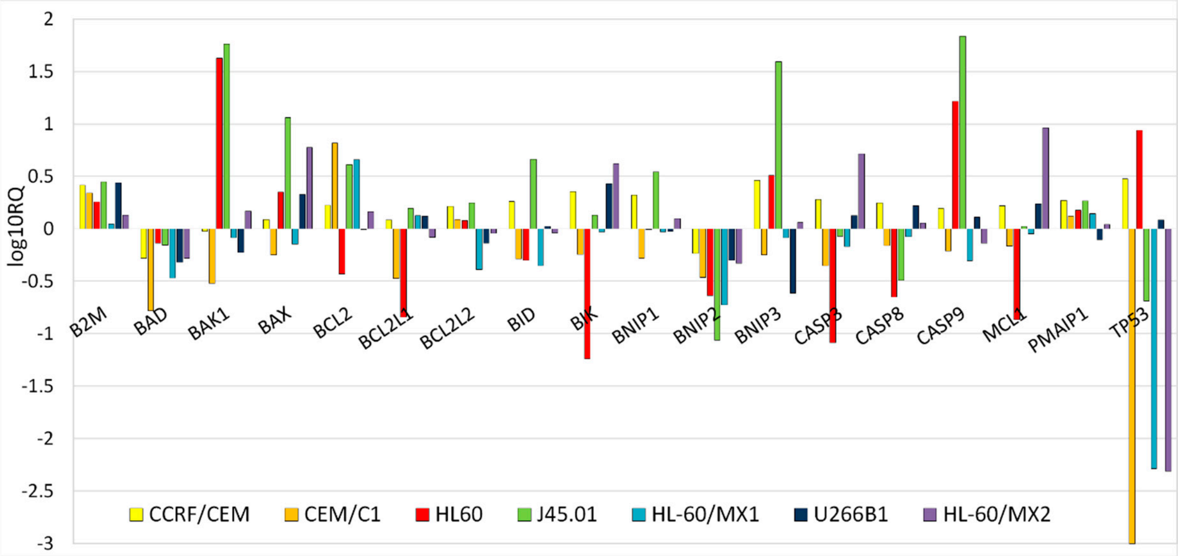

Figure S8. Expression profiles of 18 apoptosis-related genes in seven hematopoietic cell lines exposed to *Berberis thunbergii* DC. extract in concentration of 145.5 µg/mL.
